# Experimental Evaluation of QY-69: A Butyrylcholinesterase Inhibitor with Anti-Glioblastoma Efficacy

**DOI:** 10.2174/011570159X394797250701074055

**Published:** 2025-07-07

**Authors:** Kaixuan Wang, Ziyao Lu, Yuetong Duan, Siyu He, Weiping Lyu, Qinghong Liao, Qi Li, Xuehong Chen, Huanting Li

**Affiliations:** 1 Department of Neurosurgery, The Affiliated Hospital of Qingdao University, Qingdao University, Qingdao, Shandong, 266000, People’s Republic of China;; 2 School of Basic Medicine, Qingdao University, Qingdao, Shandong, 266071, People’s Republic of China;; 3 Guizhou Provincial Engineering Technology Research Center for Chemical Drug R&D, Guizhou Medical University, Guiyang, Guizhou, 550004, People’s Republic of China;; 4 State Key Laboratory of Natural and Biomimetic Drugs, School of Pharmaceutical Sciences, Peking University, Beijing, 100191, People’s Republic of China;; 5 School of Pharmacy, Qingdao University, Qingdao, Shandong, 266071, People’s Republic of China

**Keywords:** Glioblastoma, cognition protection, butyrylcholinesterase inhibitor, anti-proliferation, anti-migration, anti-invasion

## Abstract

**Introduction:**

Glioblastoma multiforme (GBM) is the most aggressive malignant primary brain tumor, characterized by poor prognosis. Moreover, cognitive impairment from the tumor and its treatments compromises patients' quality of life. Butyrylcholinesterase (BChE) inhibition enhances cognitive function. Notably, *BCHE* is overexpressed in GBM tissues; its downregulation suppresses tumor cell proliferation, migration, and invasion. This study aimed to identify a BChE inhibitor with dual functionality: anti-GBM efficacy and cognitive protection *via* modulation of neuroinflammation.

**Methods:**

QY-69 was identified from an in-house BChE inhibitor library through cytotoxicity-based screening. Its anti-GBM effects were evaluated through colony formation, wound healing, and transwell assays. Orthotopic GBM mice were treated with QY-69 orally for 15 days. Tumor progression, cognitive function (Morris water maze), and neuroinflammation (microglia and astrocyte immunofluorescence) were analyzed.

**Results:**

QY-69 exhibited significant antiproliferative activity at micromolar concentrations. *In vitro* assays demonstrated significant inhibition of GBM cell growth, migration, and invasion. Behavioral impairment in mice was improved, and the activation of astrocytes and microglia in peritumoral tissues was reduced, indicating a decrease in neuroinflammation.

**Discussion:**

QY-69 demonstrated dual therapeutic potential in GBM by inhibiting tumor progression and alleviating cognitive impairment. However, its precise molecular mechanisms remain to be elucidated. Future research should employ transcriptomic and proteomic approaches to elucidate the molecular basis of its anti-GBM activity.

**Conclusion:**

QY-69, a BChE inhibitor, exhibits potent anti-GBM activity and confers cognitive protection, positioning it as a promising dual-action therapeutic candidate. By inhibiting tumor progression and reducing neuroinflammation, it may enhance both survival and quality of life in GBM patients.

## 1 INTRODUCTION

Gliomas constitute approximately 23% of all primary brain tumors and other central nervous system tumors, with 81% being malignant [1]. Glioblastoma multiforme (GBM) is the most common malignant primary tumor within the intracranial region, comprising 61.0% of all gliomas and 51.5% of all malignant intracranial tumors [2]. Despite current multimodal therapies (maximal safe resection, radiotherapy, temozolomide (TMZ), and tumor-treating fields), median overall survival remains dismal, less than two years, with a 5-year survival rate < 10% [3-5]. However, over 90% of patients experience recurrence within two years, with limited therapeutic options at relapse [6-10]. This therapeutic impasse underscores the urgent need for innovative GBM therapies.

Beyond poor survival outcomes, GBM patients frequently develop neurocognitive deficits—including impaired attention, memory, and language function [11-13] —mediated by tumor location, volume, and growth dynamics [14-16]. Right-hemisphere tumors predominantly affect verbal memory/executive function, while left-hemisphere lesions impair attention/object recognition [17-19]. Emerging evidence highlights bidirectional neuron-tumor signaling that drives both tumor progression and cognitive dysfunction [20-22]. Concurrent symptoms (fatigue, insomnia, anorexia) and treatment-related neurotoxicity further diminish quality of life, imposing substantial psychosocial burdens [23-25]. Crucially, standard therapies exacerbate cognitive decline: 94.7% of surgical patients exhibit deficits [26], while TMZ induces hippocampal toxicity and persistent cognitive impairment [27, 28]. Consequently, developing chemotherapeutic agents with dual competence in oncologic efficacy and cognitive preservation represents a highly valuable area of fundamental research, offering broad clinical application prospects for GBM treatment.

Cholinesterase (ChE) is widely expressed in mammals, and its abnormal expression promotes cell proliferation, participating in the occurrence and development of tumors [29]. Acetylcholinesterase (AChE) is a marker of early tumor differentiation, while butyrylcholinesterase (BChE) participates in cell migration and fiber induction [30, 31]. Studies have shown that the enzymatic activity of BChE in GBM tissues is approximately twice that in normal brain tissues [32]. This significant difference suggests that inhibiting BChE activity may represent a promising therapeutic strategy for the clinical treatment of GBM. However, current knowledge of BChE in the context of GBM remains restricted, highlighting the urgent requirement for additional research to validate its potential clinical applications.

Furthermore, the function of ChE is to hydrolyze acetylcholine (ACh). In advanced Alzheimer's disease (AD), irreversible degeneration of cholinergic neurons reduces AChE activity to <10% of normal levels, during which BChE compensatorily maintains cholinergic neurotransmission [33]. BChE inhibitors exert dual therapeutic effects: (1) enhancing ACh availability *via* catalytic inhibition and (2) conferring neuroprotection against β-amyloid toxicity and oxidative stress, thereby ameliorating cognitive deficits in AD models [34, 35]. Notably, the physiological non-essentiality of BChE, as evidenced by the preserved neurological function in individuals with BChE deficiency, underscores its superior clinical translatability [36, 37]. Therefore, this study proposes a novel therapeutic paradigm: repurposing neuroprotective BChE inhibitors to identify dual-functional candidates with concurrent anti-glioblastoma activity. This strategy aims to preserve cognition while controlling tumor progression synergistically.

This study identified QY-69, a BChE inhibitor with dual anti-glioblastoma and neuroprotective properties, through Cell Counting Kit-8 (CCK-8) assays conducted on a library of 69 BChE inhibitors [35, 38, 39]. Using *in vitro* systems and GL261-Luc orthotopic models, this study quantitatively demonstrated QY-69's therapeutic effects through the inhibition of cell proliferation (CCK-8 assays and colony formation assays), suppression of migration (wound-healing assays), and modulation of invasive capacity (transwell assays). Bioluminescence imaging quantified tumor regression, while Morris water maze (MWM) tests and peritumoral immunofluorescence profiling confirmed neurocognitive preservation and modulation of the inflammatory microenvironment, respectively. The findings of this study established BChE inhibition as a promising strategy for GBM treatment that concurrently addressed tumor progression and neurocognitive preservation, advocating for therapeutic paradigms integrating neuroprotection with conventional survival metrics.

## 2 MATERIALS AND METHODS

### 2.1 Compound Synthesis

Following compound screening, QY-69 was identified as the lead compound for preclinical evaluation [35, 38, 39]. A Large-scale synthesis of QY-69 was conducted following published methodologies, with reaction monitoring *via* thin-layer chromatography. Structural verification was achieved through ^1^H-NMR (Fig. **S1**). ^1^H NMR (400 MHz, DMSO-*d_6_*) δ 10.15 (s, 1H), 7.87 (d, *J* = 8.0 Hz, 1H), 7.82 (d, *J* = 8.1 Hz, 1H), 7.66 (dd, *J* = 8.3, 2.7 Hz, 1H), 7.55-7.35 (m, 3H), 7.30-7.21 (m, 1H), 6.99 (s, 2H), 5.65 (s, 2H).

### 2.2 Cell Culture

The glioma cell lines (U251 and C6) were obtained from the Cell Culture Center of the Institute of Basic Medical Sciences, Chinese Academy of Medical Sciences (Shanghai, China). GL261 and GL261-luc cells were purchased from Ubigene Biosciences Co., Ltd. (China). Dulbecco's Modified Eagle Medium (DMEM), phosphate-buffered saline (PBS), trypsin, and CCK-8 were procured from KeyGen BioTECH Co., Ltd. (China). Cells were maintained in DMEM supplemented with 100 U/mL penicillin, 100 µg/mL streptomycin, and 10% fetal bovine serum (FBS), and incubated at 37°C in a humidified 5% CO_2_ atmosphere.

### 2.3 Animals

C57BL/6 male mice (5-6 weeks, 17-20 g) and KM male mice (3-4 weeks, 22-25 g) were obtained from Huafukang Biotechnology (China). Animals were housed in specific pathogen-free facilities, maintained at 23 ± 2°C with 40-60% humidity under a 12/12-hour light/dark cycle, with ad libitum access to food and water. Daily monitoring of mice included assessments of weight and behavior. Euthanasia was conducted under the following conditions: 1) a weight loss exceeding 20%; 2) an inability to eat or drink; 3) neurological or respiratory dysfunction persisting for more than 24 hours.

### 2.4 Cytotoxicity Experiments

Cytotoxicity tests were assessed using Cell Counting Kit-8 (CCK-8) assays [40]. Cells in their exponential growth phase were seeded into 96-well plates (5×10^3^ cells/well). After 24 hours of adhesion, the medium was replaced with serially diluted QY-69. Following 24-, 48-, or 72-hour treatment, 10 μL of CCK-8 reagent was added per well and incubated for 2 hours at 37°C. The 96-well plate was then placed in a microplate reader, and the optical density (OD) was measured at 450 nm. The absorbance of the blank control group was set as 100%, and the cell viability (%) was calculated using the following formula:

Cell viability (%) = OD _(compound-treated group)_ / OD _(blank-control group)_ × 100%.

### 2.5 The Colony Formation Assay

Glioma cell lines (U251 and GL261) in the logarithmic growth phase were seeded in 6-well plates (1,000 cells/well) [41]. The monolayer cultures were then treated with two different concentrations of QY-69 for 10 days, with medium replacement every 72 hours. Following incubation, cells were washed with PBS and fixed with 4% paraformaldehyde (Biosharp) for 20 minutes. The colonies were subsequently stained with 0.1% crystal violet dye (Biosharp) for visualization. The above experiment was repeated three times, and the number of purple spots in each well, which represented the number of colonies formed, was imaged and recorded.

### 2.6 Wound-healing Assay

U251 and GL261 cells were seeded in 6-well plates (2×10^5^ cells/well) and cultured to ~90% confluency [42]. Uniform linear scratches were created in the monolayer using a sterile pipette tip. After removing the medium and washing with PBS to clear debris, cells were maintained in low-serum medium (1% FBS) containing QY-69. Phase-contrast images were acquired at 0 and 48 hours post-treatment using an inverted microscope. The cell migration rate was calculated as: Migration rate (%) = (1 - area of the scratch after migration/initial scratch area) × 100%.

### 2.7 Transwell Assay

Cells (U251 and GL261) were serum-starved in basal medium for 6 hours [43]. The transwell inserts (8 μm pores; Corning) were pre-coated with 2% Matrigel matrix. Serum-starved cells (5×10^4^ cells/well) were seeded in upper chambers containing serum-free medium, while lower chambers were loaded with complete medium containing 10% FBS as a chemoattractant. Following a 48-hour incubation at 37°C, migrated cells on the lower membrane surface were fixed with 4% paraformaldehyde (Beyotime) for 15 minutes and stained with 0.1% crystal violet (Beyotime). Non-migratory cells were mechanically removed using cotton swabs. Moreover, migratory cells in randomly selected fields were imaged under an inverted microscope and quantified.

### 2.8 Acute Toxicity Evaluation *in Vivo*

QY-69 was formulated in a vehicle (10% DMSO, 40% PEG-300, 50% saline). After overnight fasting and simple randomization, seven KM male mice (3-4 weeks, 22-25 g) received a single oral gavage of QY-69 (1 g/kg), with seven vehicle-treated controls receiving equivalent volumes [44]. Over the two weeks following administration, daily monitoring of body weight, survival status, and behavioral changes was conducted. At the end of the observation period, survivors were euthanized *via* pentobarbital overdose for histopathological analysis. Tissues were fixed in 4% paraformaldehyde (24 hours), paraffin-embedded, and sectioned into 5-μm slices. Sections were processed through standard dewaxing and hematoxylin and eosin (H&E) staining protocols for histopathological evaluation.

### 2.9 Establishment of Orthotopic GBM Models

An orthotopic GBM mouse model was established using a stereotaxic apparatus (RWD Life Science, Cat# 68805). C57BL/6 male mice (5-6 weeks, 17-20 g) were anesthetized with pentobarbital sodium (50 mg/kg, i.p.), followed by a 2-cm sagittal incision exposing the bregma. A 1-mm burr hole was drilled 2 mm lateral and 2 mm posterior to bregma [45]. GL261-Luc cells (2×10^5^ cells in 2 μL PBS) were injected 3 mm below the cortical surface at 1 μL/min [46]. The injection cannula remained *in situ* for 10 minutes post-injection to minimize reflux before surgical closure.

Tumor establishment was longitudinally monitored *via* bioluminescence imaging (IVIS Lumina XRMS III; PerkinElmer) at days 4, 11, and 18 post-implantation following an i.p. administration of D-luciferin (150 mg/kg, Mreda) [47]. Quantitative analysis of bioluminescent signals was performed using Living Image 4.5 software (PerkinElmer) with region-of-interest analysis targeting the cranial compartment.

### 2.10 GL261 Model Treatment

C57BL/6 male mice (5-6 weeks, 17-20 g) were randomized into four experimental groups (n = 5/group [39]): control group (oral gavage vehicle solution), sham-operation group (intracerebral injection PBS + oral gavage vehicle solution), GL261-Luc model group (intracerebral injection GL261-Luc cells + oral gavage vehicle solution) and GL261-Luc + QY-69 (intracerebral injection GL261-Luc cells + oral gavage 30 mg/kg/day QY-69), followed by baseline verification of tumor burden (*via* bioluminescent imaging) and body weight to confirm intergroup homogeneity prior to treatment initiation. QY-69 was formulated as described in Section 2.8 The control, sham, and GL261-Luc model groups received equivalent vehicle administration. Tumor progression was longitudinally monitored *via* bioluminescence imaging on days 11 and 18 using the Living Image 4.5 software (PerkinElmer) for volumetric analysis.

### 2.11 Morris Water Maze Test

The behavioral capacity was assessed using a Morris water maze (MWM) test [35]. The maze consisted of a circular pool (60 cm diameter × 45 cm height) filled to a depth of 30 cm with water maintained at 25°C. A hidden platform (6 cm radius) was positioned 1 cm below the water surface in a target quadrant. From days 12 to 17 post-tumor inoculation, mice were subjected to daily acquisition trials consisting of two 60-second sessions per day. On day 18, a probe trial was conducted with the platform removed. To ensure that any observed deficits were not due to motor or visual impairments, visible platform tests (platform 0.5 cm submerged) were conducted on day 1, prior to the initiation of hidden platform training on days 2 to 6. Escape latency (time to platform) and escape path were recorded using the Panlab SMART 3.0 tracking system. Mice failing to locate the platform within 60 seconds were guided to it and allowed 30 seconds of spatial orientation.

### 2.12 Tumor Specimens

On day 18 of pharmacodynamic evaluation, mice were anesthetized with 50 mg/kg pentobarbital sodium (i.p.) and fixed *via* cardiac perfusion. Following surgical decapitation, intracranial tumors were excised. Tumor volume was calculated using the ellipsoid formula [48]:


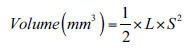


where “L” represented the longest diameter and “S” represented the shortest diameter.

### 2.13 Immunofluorescence Staining

Brain tissues were harvested and immediately fixed in 4% paraformaldehyde (4°C, 24 hours). Sequential cryoprotections were performed through graded sucrose immersion (15% to 30%) until tissue sedimentation. Dehydrated tissues were embedded in optimal cutting temperature compound, flash-frozen in liquid nitrogen, and sectioned into 10-μm slices using a Leica CM1900 cryostat. For immunofluorescence, sections were incubated with primary antibodies at 4°C overnight: Rabbit anti-IBA1 (1:300, Servicebio) and Mouse anti-GFAP (1:600, Servicebio). After PBS washes, sections were stained with secondary antibodies for 50 minutes (light-protected, RT): Cy3-conjugated Goat anti-Rabbit IgG (1:300), Alexa Fluor 488-conjugated Goat anti-Mouse IgG (1:400). Nuclear counterstaining was performed using DAPI (10 minutes, RT). Fluorescence imaging was conducted using a 3D HISTECH PANNORAMIC digital pathology system.

### 2.14 Data and Statistical Analysis

All *in vitro* experiments were conducted with ≥ 3 biological replicates. Data are expressed as mean ± SEM and analyzed using GraphPad Prism 8.0. Statistical comparisons were performed as follows: two-group analyses utilized Student's t-test (two-tailed, equal variances) or Welch's t-test (unequal variances), multi-group comparisons employed one-way ANOVA with Dunnett's post hoc test. Statistical significance was defined as *P* < 0.05. Blinding could not be implemented during treatment; whenever possible, it was done in a blinded fashion.

## 3 RESULTS

### 3.1 Identification of BChE Inhibitor with Anti-GBM Activity

**Fig. (1):**
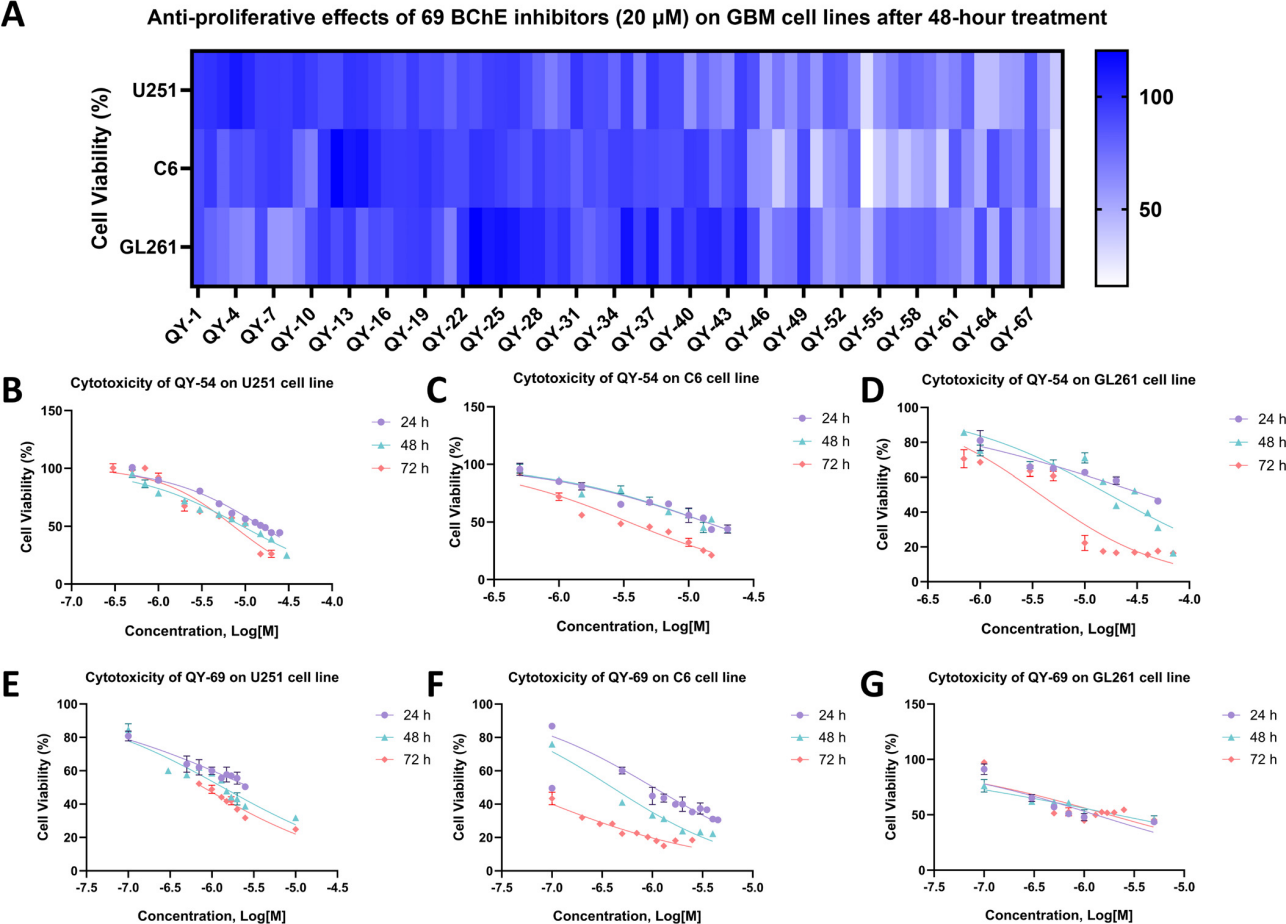
The antiproliferative ability of BChE inhibitors in an in-house database. (**A**) The antiproliferative capacity of 69 BChE inhibitors on three GBM cell lines (U251, C6, GL261). (**B-G**) The cell proliferation inhibition curve of QY-54 and QY-69 on three GBM cell lines (U251, C6, GL261). The Y-axis showed cell viability (%) (CCK-8-detected survival rate); calculation details are in Section 2.4

To identify anti-GBM compounds with neuroprotective effects, this study conducted anti-proliferation assays using an in-house library of BChE inhibitors on three different GBM cell lines: human U251 cells, rat C6 cells, and murine GL261 cells. Firstly, this study evaluated the antiproliferative ability of 69 BChE inhibitors in the in-house database at a concentration of 20 µM for 48 hours. As the result (Fig. **1A**; Fig. **S2A-C**), two compounds, QY-54 (human BChE IC_50_ = 155 ± 56.4 nM) and QY-69 (human BChE IC_50_ = 62.2 ± 22.1 nM) (Fig. **S3**) showed more than 50% inhibition on all three GBM cell lines. To further evaluate the anti-glioma efficacy of compounds QY-54 and QY-69, the Cell Counting Kit-8 (CCK-8) assay was subsequently employed to determine the half-maximal inhibitory concentration (IC_50_) on U251, C6, and GL261 GBM cells at 24, 48, and 72 hours. As illustrated in Fig. (**1B**-**G** and Table **1**), compounds QY-54 and QY-69 effectively curtailed the growth of the three glioma cell lines with IC_50_ values consistently below 5 μM. The inhibitory effect of compounds QY-54 and QY-69 on the proliferation of three cell lines is time-dependent and concentration-dependent. The *in vitro* anti-GBM activity of both compounds is superior to that of the positive control drug TMZ (Table **1**), especially for compound QY-69. Therefore, compound QY-69 was selected as the preferred molecule for subsequent experiments.

### 3.2 *In Vitro* Sustained Antiproliferative Effects Against GBM Cells

**Fig. (2):**
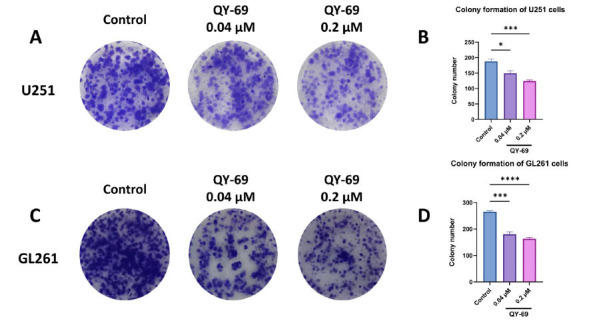
The inhibitory effect of compound QY-69 on the colony formation of U251 and GL261 cells. (**A** and **B**) Representative images of colony formation in U251 (**A**) and GL261 (**B**) cells with QY-69 or vehicle. (**C** and **D**) Quantitative analysis of colony numbers in U251 (**C**) and GL261 (**D**) cells. The Y-axis represented the number of clones (*i.e*., the clone colonies observed in the experiment). All tests were expressed as the mean ± SEM of three separate experiments. Significant differences are denoted as follows: *P < 0.05, *** *P* < 0.001, **** *P* < 0.0001 *vs.* Control group.

The long-term antiproliferative effects of QY-69 were assessed in GBM cell lines (U251, GL261) using colony formation assays. The visual outcomes and numerical data are illustrated in Figs. (**2A**-**D**). The vehicle-treated U251 and GL261 cells formed 265.2 and 188.0 colonies, respectively, after 10 days (Figs. **2C** and **D**). QY-69 treatment significantly suppressed colony formation, demonstrating significant inhibitory activity.

### 3.3 *In Vitro* Migration and Invasion Inhibitory Capability Evaluation Against GBM Cells

To assess the anti-migratory and anti-invasive effects of QY-69 on GBM, we performed wound healing and transwell invasion assays. To isolate migration/invasion phenotypes from proliferation effects, inhibitory concentrations (0.02 μM, 0.1 μM) were selected based on prior proliferation data (U251/GL261 IC_50_ values).

**Fig. (3):**
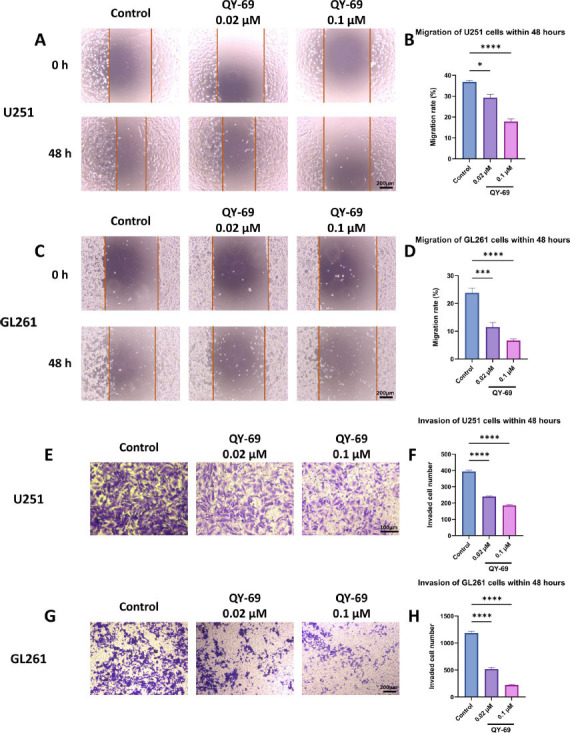
The inhibitory activity of QY-69 on the migration and invasion of U251 and GL261 cells. (**A** and **B**) Representative scratch assay images of U251 (**A**) and GL261 (**B**) cells 48 hours post-treatment with QY-69. Scale bars: 200 μm. (**C** and **D**) Quantitative analysis of relative migration rates for U251 (**C**) and GL261 (**D**) cells. The Y-axis showed cell migration rate (%) (scratch test proportion); see calculation details in 2.6 (**E** and **F**) Transwell invasion assay images of U251 (**E**) and GL261 (**F**) cells treated with QY-69. Scale bars: 100 μm (**E**), 200 μm (**F**). (**G** and **H**) Quantified invasive cell counts for U251 (**G**) and GL261 (**H**) cells. All tests were expressed as the mean ± SEM of three separate experiments. Significant differences are denoted as follows: *P < 0.05, ***P < 0.001, ****P < 0.0001 *vs.* Control group.

Wound healing assays demonstrated significant inhibition of GBM cell migration by QY-69, compared to vehicle-treated groups (Figs. **3A**-**D**). Subsequently, transwell invasion experiments were performed on GL261 and U251 cells to assess the impact of QY-69 on its ability to invade through Matrigel matrix gel and basement membrane. Transwell invasion analysis revealed potent suppression of invasiveness by QY-69, compared with the control groups (Figs. **3E**-**H**).

### 3.4 Initial *In Vivo* Safety Evaluation

**Fig. (4):**
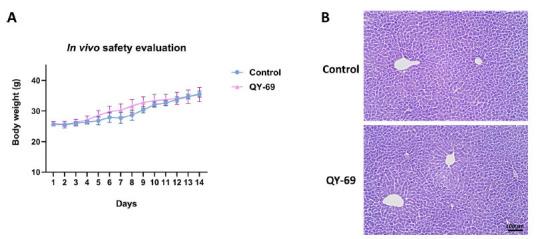
Preliminary *in vivo* safety evaluation of QY-69. (**A**) Body weight trajectories of mice following single oral administration of QY-69 (1 g/kg) over a 14-day observation period. (**B**) Representative H&E-stained liver sections from experimental groups after 14 days (n = 7). Scale bar: 100 μm.

Drug safety evaluation constitutes a critical phase in pharmaceutical development. Acute toxicity assessment, involving the monitoring of weight fluctuations, mortality, and behavioral changes within 14 days post-single high-dose administration, serves as a cornerstone for preclinical safety profiling. In this study, mice receiving a single oral dose of QY-69 (1 g/kg) exhibited comparable weight gain trajectories to vehicle controls throughout the 14-day observation period, with no mortality or aberrant behaviors observed (Fig. **4A**). Histopathological analysis, performed *via* hematoxylin and eosin (H&E) staining, revealed a preserved hepatic architecture across central lobular, midzonal, and periportal regions, with no evidence of necrosis or steatosis (Fig. **4B**).

### 3.5 *In Vivo* Anti-GBM Evaluation

**Fig. (5):**
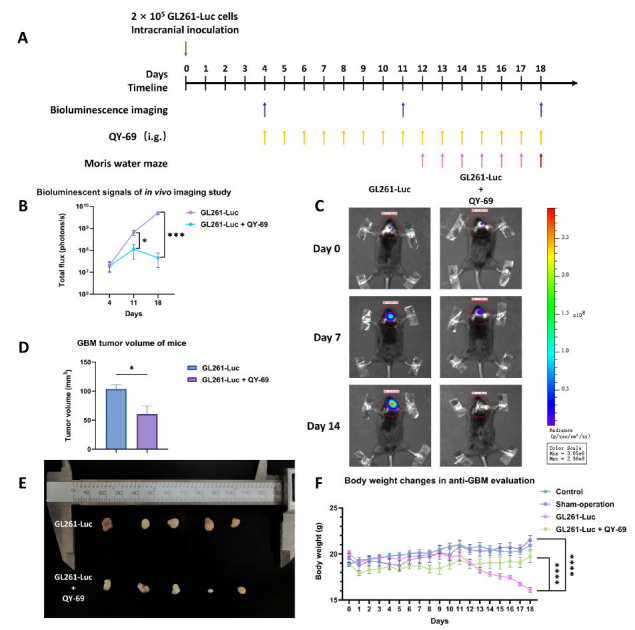
*In vivo* anti-GBM efficacy of compound QY-69. (**A**) Schedule of the *in vivo* pharmacodynamic experiment. (**B**) Bioluminescence quantification analysis of intracranial tumor *in vivo* imaging across all groups of tumor-bearing mice. The Y-axis showed the total photon count (photons/s) in bioluminescence imaging. (**C**) Representative bioluminescence imaging results from mice. (**D**) GBM tumor volume measurements from mice after 15 days of treatment. (**E**) Images of GBM tumors from mice after 15 days of treatment. (**F**) Body weight change curves of each group in the pharmacodynamic experiment process. (n = 5). *P < 0.05, ***P < 0.001, **** *P* < 0.0001 *vs.* GL261-Luc group.

As glioma is an intracranial tumor, this study established an orthotopic GL261-Luc model in C57BL/6J mice to recapitulate clinical disease progression (Fig. **5A**). Mice were randomized into different groups, and their tumor size and body weight were then monitored to ensure there were no significant differences at the beginning of the experiment (Fig. **5B**). From day 4, treatment groups received daily oral gavage of QY-69 (30 mg/kg), whereas the other groups received equivalent vehicle administration.

Serial *in vivo* imaging on days 11 and 18 post-inoculation revealed significant tumor suppression in QY-69-treated mice compared to the GL261-Luc group (Figs. **5B** and **5C**). On the 18^th^ day after tumor inoculation, the mice were immediately euthanized, and the tumor volume was measured (Figs. **5D** and **5E**). The results were consistent with those obtained from bioluminescence imaging. It was also found that the body weights of both the sham and GL261-Luc + QY-69 groups were significantly higher than those of the GL261-Luc group (Fig. **5F**). Concurrently, the GL261-Luc group exhibited progressive weight loss, whereas QY-69-treated mice maintained stable body mass (Fig. **5F**). These findings indicate that QY-69 treatment improves prognosis by effectively slowing tumor growth.

### 3.6 The Impact of QY-69 Treatment on Orthotopic Glioma Mice Performance in MWM

**Fig. (6):**
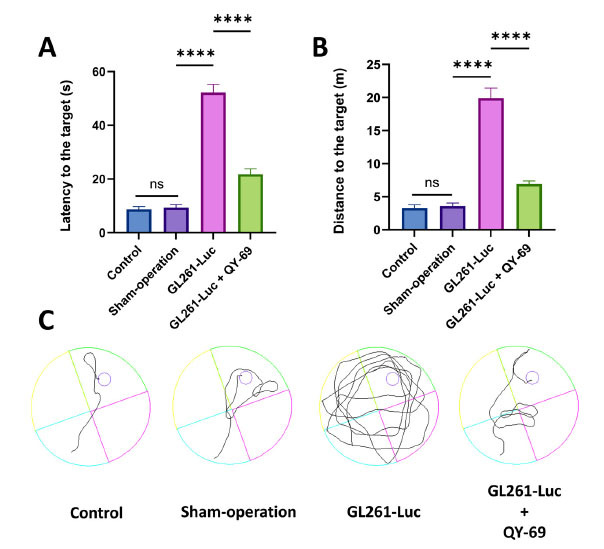
*In vivo* behavioral evaluation. (**A**) Latency to reach the platform for each group of mice in the MWM. The Y-axis showed the time it took tumor-bearing mice to swim from the water maze start to the hidden platform. (**B**) Distance traveled by each group of mice to reach the platform in the MWM. The Y-axis showed the distance that tumor-bearing mice swim from the water maze start to the hidden platform. (**C**) Representative movement trajectories of each group of mice in the MWM. (n = 5). ns, no significance between sham-operation and control group, ****P < 0.0001 *vs.* GL261-Luc group.

Developing anti-GBM agents with cognition-protective potential is of vital importance for GBM patients. To examine the impact of compound QY-69 on cognitive performance in orthotopic glioma-bearing mice, Morris water maze tests (MWM) were initiated on the eighth day of treatment, and the behavioral performance of mice in different groups was assessed on the final day (Fig. **5A**). Comparing the animal behaviors in the control and sham-operation groups, this study concludes that the surgical procedure did not significantly impair cognitive performance (Figs. **6A**-**C**). In comparison to the mice behaviors in the sham-operation group, those in the GL261-Luc group exhibited more pronounced cognitive deficits, characterized by increased escape latency, longer escape distances, and more complex escape trajectories. Notably, treatment with compound QY-69 resulted in remarkably reduced escape times and distances to reach the platform, and simplified escape trajectories.

### 3.7 The effects of QY-69 on Microglia and Astrocytes in Peritumoral Brain Tissue

**Fig. (7):**
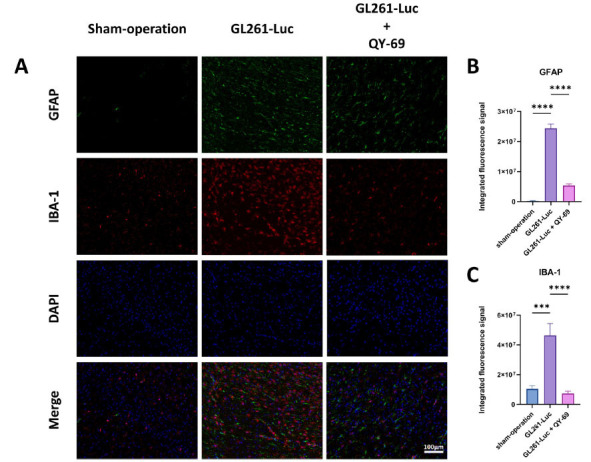
Effects on astrocytes and microglia in the peritumoral brain region. (**A**) Immunofluorescence staining images of GFAP (green), IBA-1 (red), and DAPI (blue) in the brain region surrounding the tumor. (scale bar 100 µm). (**B** & **C**) Quantitative assessment of GFAP^+^ astrocytes and IBA-1^+^ microglia in the peritumoral brain region by immunofluorescence staining. The Y-axis showed integrated fluorescence density for the same-sized areas. (n = 5). ***P < 0.001; ****P < 0.0001 *vs.* GL261-Luc group.

Emerging evidence indicates that chronic neuroinflammation triggered by the glioma microenvironment contributes significantly to the pathogenesis of neuropsychiatric disorders [49]. Notably, peritumoral brain regions exhibit accelerated aging phenotypes including mitochondrial dysfunction, chronic neuroinflammation, and impaired protein homeostasis [50]. These areas also manifest AD-like pathologies such as lipofuscin deposition, tau hyperphosphorylation, and oxidative DNA damage [51]. Given the recognized role of BChE inhibitors in counteracting neuroinflammation and preserving neuronal function [34], this study investigated QY-69's effects on peritumoral glial cells. Immunofluorescence analysis showed elevated GFAP^+^ astrocytes and IBA-1^+^ microglia in GL261-Luc mice compared to the sham-operation group, indicating neuroinflammatory activation (Figs. **7A**-**C**). QY-69 significantly reduced both GFAP^+^ and IBA-1^+^ cell densities in peritumoral regions (Figs. **7A**-**C**), demonstrating its capacity to suppress inflammatory glial activation and providing additional evidence for the potential therapeutic value of such inhibitors in mitigating neuroinflammation.

## 4 DISCUSSION

GBM, the most common malignant primary brain tumor in adults, is characterized by aggressive growth, therapeutic resistance, and inevitable recurrence patterns [52]. These characteristics collectively result in a poor prognosis for GBM patients, with a mOS of less than two years and a 5-year survival rate below 10% [5, 53]. Patients with slower tumor growth would transfer affected cognitive function to unaffected brain regions over time, indicating that the impact of GBM on the brain is overall [54].

In addition to the tumor itself, surgical procedures, radiation therapy, and chemotherapy can also cause significant cognitive harm [55-57]. Recently, research on GBM has highlighted the bidirectional signaling between tumors and neurons [58]. This signaling not only promotes tumor proliferation and invasion but also contributes to cognitive impairments in patients [20, 59]. Wang *et al.* found that disruption type gliomas are associated with the IDH wild-type, and patients with destructive gliomas are more likely to experience postoperative cognitive impairment [60]. This finding is consistent with the research of Bunevicius *et al.* [61]. In recent years, studies have clearly shown that chemotherapy-mediated neurotoxicity is becoming increasingly important [62]. Even after stopping treatment, impaired attention, executive ability, and memory cannot be salvaged, thereby affecting quality of life [63]. A study shows that after receiving standard treatment with the first-line chemotherapy drug TMZ for GBM, 25% of patients will experience grade 3-4 cognitive impairment, especially in elderly patients with a higher risk of toxicity [64].

Therefore, one of the main treatment goals for GBM is to reduce the incidence of complications, restore or preserve neurological function, and prolong the effective daily activity time of patients as much as possible [65, 66]. Due to the very short life expectancy of GBM patients, certain “quality of life”-related issues, such as improving patients' cognitive function, emotional well-being, and nutritional status, are of paramount importance for patients and their caregivers [67, 68]. This clinical reality underscores the critical need for developing chemotherapeutic agents that synergistically target tumor eradication and neural preservation [69].

The cholinesterase family, particularly BChE, has emerged as a strategic target in oncology. It is noteworthy that BChE exhibits characteristic overexpression in various malignant tumors, including breast cancer, oral cancer, prostate cancer, gastric cancer, ovarian cancer, and GBM [70-78]. Mechanistically, *BCHE* silencing in neuroblastoma models suppresses N-Myc proto-oncogene expression while inhibiting invasion-associated tyrosine kinases (C-Ros oncogene 1, tropomyosin-related kinase B, and leukocyte tyrosine kinase) [79]. Given the elevated expression of BChE in GBM tissues, where enzymatic activity is approximately twice that observed in normal brain tissues, the inhibition of ChE activity may present potential therapeutic advantages for the treatment of GBM [32, 75, 76]. More over, cholinesterase inhibition elevates synaptic cetylcholine levels, potentially counteracting treatment-related cognitive deficits. This mechanism has been clinically validation in donepezil, an acetylcholinesterase inhibitor that confers modest improvements in several cognitive functions among patients with pretreatment severe cognitive impairment [80].

BChE inhibitors offer additional therapeutic advantages through β-amyloid disaggregation and the mitigation of oxidative stress, demonstrating neuroprotective efficacy in Alzheimer's disease models [81]. Furthermore, owing to their distinct safety advantages, this class of inhibitors has significant translational potential in clinical applications [82]. Meanwhile, there is a growing focus among researchers on drug molecules that exhibit both cognitive-protective and anti-tumor properties within the tumor context. It has been found that notopterol, a compound extracted from traditional Chinese medicine, has the potential to ameliorate cognitive impairments by suppressing inflammation in the brain tissue surrounding GBM [45].

Employing a screening strategy for BChE inhibitors with anti-GBM activity, this study evaluated an in-house compound library against three GBM cell lines (U251, C6, GL261) using CCK-8 assays. This identified QY-54 and QY-69 as lead candidates with potent antiproliferative effects. Subsequent IC_50_ assays revealed QY-69's superior efficacy compared to QY-54 and the first-line agent TMZ. Functional assays demonstrated QY-69's capacity to inhibit clonogenicity, migration, and invasion in U251 and GL261 models - phenotypes consistent with the effects of BCHE knockdown reported in gastric cancer [74]. Importantly, acute toxicity studies revealed QY-69 exhibited no significant weight fluctuations or hepatic histopathological changes, indicating favorable *in vivo* tolerability.

To validate the *in vivo* anti-tumor efficacy, this study established orthotopic GL261-Luc models in C57BL/6 mice [46]. Bioluminescence imaging revealed significantly smaller intracranial tumors in QY-69-treated mice versus controls, which was further confirmed through post-mortem volumetric analysis of excised tumor tissues. Cognitive assessments, as measured by MWM testing, demonstrated a marked behavioral improvement in QY-69-treated tumor-bearing mice after 15-day treatment. Immunofluorescence analysis showed decreased activation of tumor-associated GFAP^+^ astrocytes and IBA-1^+^ microglia, consistent with reported mechanisms linking op to glioma-related cognitive deficits [45]. These results suggest that QY-69 may play a role in mitigating neuroinflammation in the areas surrounding tumors.

This study has several limitations that warrant further investigation. The anti-glioma mechanism of QY-69 remains incompletely understood, particularly regarding its specific effects on tumor cell cycle regulation, apoptotic signaling pathways, and epigenetic modifications. Future research should employ transcriptomic and proteomic analyses of pre- versus post-QY-69 treatment specimens, combined with immunoprecipitation-mass spectrometry mapping of QY-69-associated protein-RNA interactomes. These approaches would help elucidate the molecular circuitry underlying QY-69-mediated suppression of GBM malignancy.

## CONCLUSION

In this study, the compound QY-69 was identified as having the best anti-GBM activity from 69 compounds in our in-house database. It exhibited antiproliferative activity on GBM cells from different species sources at a micromolar level, significantly inhibiting tumor cell growth, migration, and invasion. In the *in vivo* anti-GBM experiments, compound QY-69 significantly slowed down the growth rate of GBM tumors. After administration of QY-69, the behavioral impairment of mice was significantly improved, and the activation of astrocytes and microglia in the peritumoral tissues was reduced, indicating that BChE inhibition can reduce neuroinflammation in the peritumoral tissues. In summary, the BChE inhibitor QY-69 has the potential to resist GBM and improve the cognition of GBM patients; however, its detailed anti-GBM mechanism still needs to be further explored.

## Figures and Tables

**Table 1:** Comparison of QY-54, QY-69, and TMZ effects on GBM cell proliferation (U251, C6, GL261)*^a^.*

**Cell Lines**	**Time (h)**	**QY-54 *^b^***	**QY-69 *^b^***	**TMZ *^c^***
U251	24 h	15.48 ± 1.14	2.89 ± 0.70	94.27 ± 2.43
	48 h	9.32 ± 1.02	1.36 ± 0.22	89.03 ± 2.66
	72 h	7.37 ± 1.10	0.79 ± 0.11	83.13 ± 3.22
C6	24 h	13.54 ± 2.12	1.03 ± 0.12	94.73 ± 2.31
	48 h	13.59 ± 2.12	0.40 ± 0.06	81.05 ± 2.04
	72 h	3.46 ± 0.50	0.04 ± 0.01	64.52 ± 2.04
GL261	24 h	37.79 ± 15.67	1.30 ± 0.49	98.26 ± 0.69
	48 h	17.23 ± 3.48	2.12 ± 0.84	79.31 ± 1.38
	72 h	3.78 ± 0.80	1.78 ± 0.78	50.27 ± 1.98

*^a^*All tests were expressed as the mean ± SEM of three separate experiments.

*^b^*IC_50_ value (µM).

*^c^*Survival rate at 100 µM.

## Data Availability

All data generated or analyzed during this study are included in this published article.

## LIST OF ABBREVIATIONS

Ach: Acetylcholine
AChE: Acetylcholinesterase
AD: Alzheimer’s Disease
BChE: Butyrylcholinesterase
CCK-8: Cell Counting Kit-8
ChE: Cholinesterase
DAPI: 4’,6-diamidino-2-phenylindole
DMEM: Dulbecco’s Modified Eagle’s Medium
FBS: Fetal Bovine Serum
GBM: Glioblastoma Multiforme
H&E: Hematoxylin and Eosin
MWM: Morris Water Maze
PBS: Phosphate Buffer Solution
TMZ: Temozolomide
