# Supplementary material for: Experimental Evaluation of QY-69: A Butyrylcholinesterase Inhibitor with Anti-Glioblastoma Efficacy
Source: Curr Neuropharmacol. 2025 Jul 7;24(5):725–39. doi: 10.2174/011570159X394797250701074055 (PMC13270083; doi:10.2174/011570159X394797250701074055)
Supplement: Supplementary file 1 [file CN-24-5-725_SD1.pdf]

Supplementary Material

Experimental Evaluation of QY-69: A Butyrylcholinesterase Inhibitor with Anti-Glioblastoma Efficacy

Kaixuan Wang[1,2,#], Ziyao Lu[2,#], Yuetong Duan[2], Siyu He[3], Weiping Lyu[4], Qinghong Liao[5], Qi Li[2], Xuehong Chen[2,*] and Huanting Li[1,*]

[1]*Department of Neurosurgery, The Affiliated Hospital of Qingdao University, Qingdao University, Qingdao, 266000, Shandong, People's Republic of China;* [2]*School of Basic Medicine, Qingdao University, Qingdao, 266071, Shandong, People's Republic of China;* [3]*Guizhou Provincial Engineering Technology Research Center for Chemical Drug R&D, Guizhou Medical University, Guiyang, 550004, People's Republic of China;* [4]*State Key Laboratory of Natural and Biomimetic Drugs, School of Pharmaceutical Sciences, Peking University, Beijing, 100191, People's Republic of China;* [5]*School of Pharmacy, Qingdao University, Qingdao, 266071, Shandong, People's Republic of China*

1875-6190/26 $65.00+.00 **© 2026 Bentham Science Publishers**

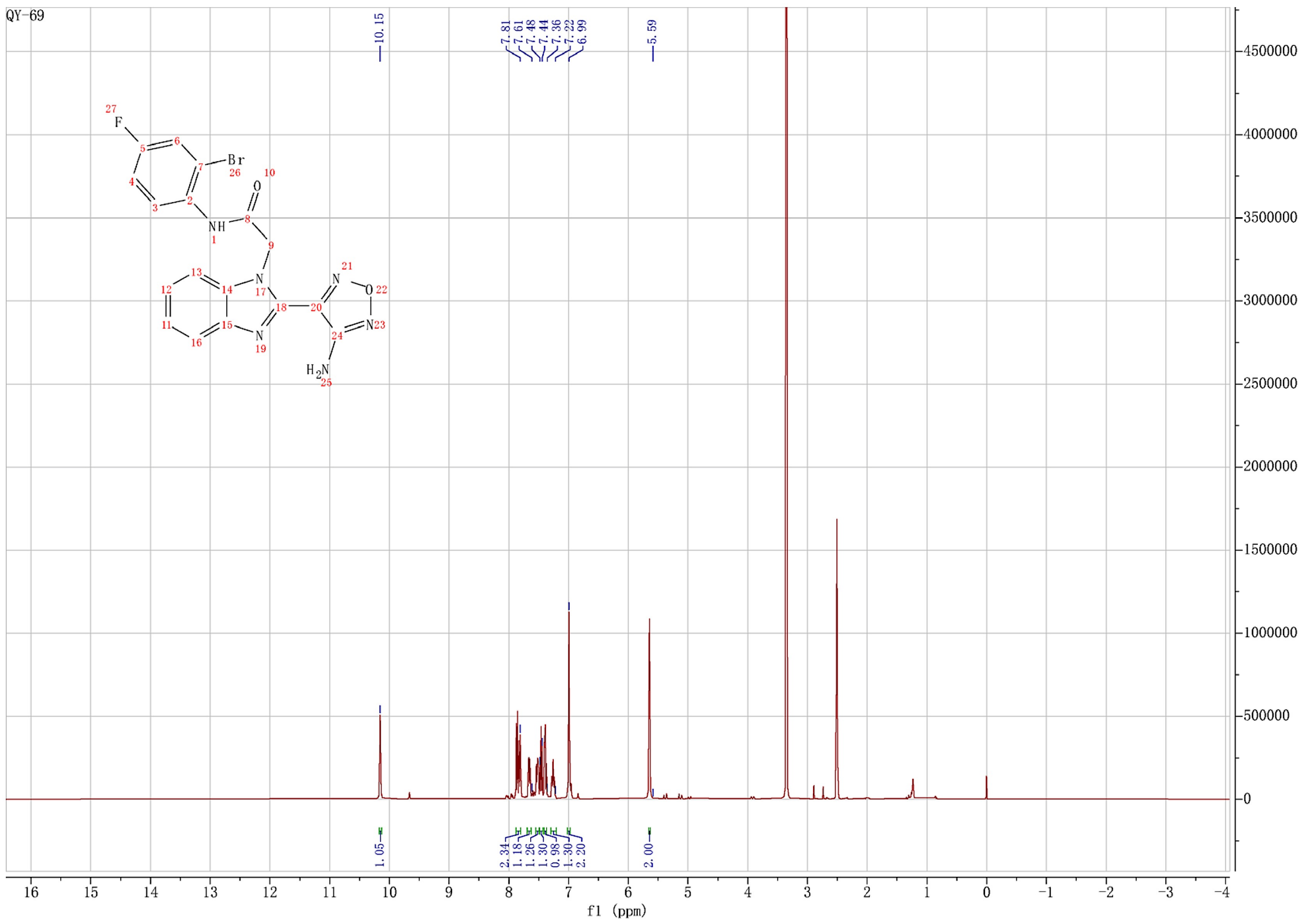

Fig. (S1). The 1H-NMR characterization of **QY-69**.

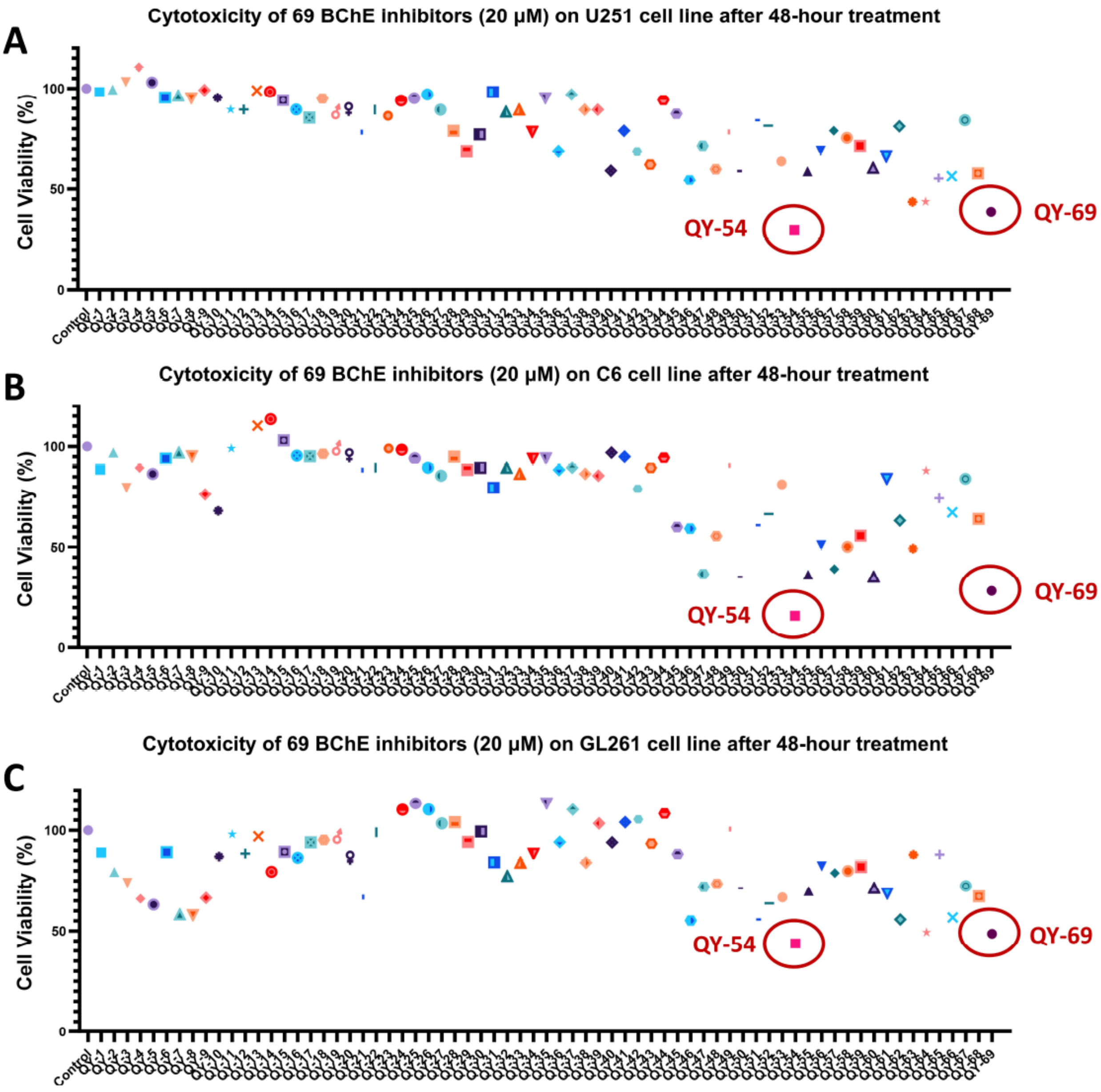

Fig. (S2). The anti-proliferative capacity of 69 BChE inhibitors on three GBM cell lines (U251, C6, GL261).

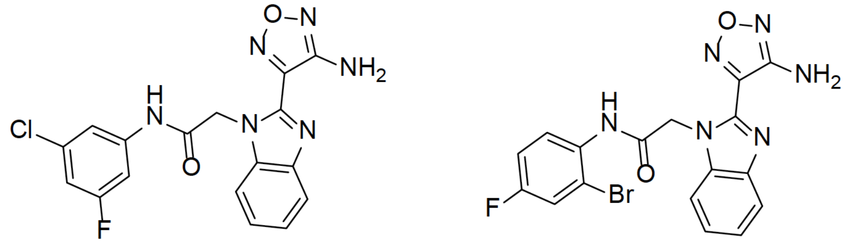

QY-54 **QY-69**

Fig. (S3). Chemical structures of **QY-54** and **QY-69**.
